# Association of Internet Addiction with Emotional and Behavioral Characteristics of Adolescents 

**Published:** 2020-01

**Authors:** Mohammad Effatpanah, Mohammad Moharrami, Gilda Rajabi Damavandi, Mahdi Aminikhah, Mohammad Hosein Nezhad, Farnaz Khatami, Tara Arjmand, Heliya Tarighatnia, Mir Saeed Yekaninejad

**Affiliations:** 1 School of Medicine, Ziaeian Hospital, International Campus, Tehran University of Medical Sciences, Tehran, Iran.; 2 School of Dentistry, Tehran University of Medical Sciences, Tehran, Iran.; 3 Ziaeian Hospital, Tehran University of Medical Science, Tehran, Iran.; 4 Department of Epidemiology and Biostatistics, School of Public health, Tehran University of Medical Sciences, Tehran, Iran.; 5 Farhangiyan University, Tehran, Iran.; 6 Family Medicine Department, Ziaeian Hospital, Tehran University of Medical Sciences, Tehran, Iran.

**Keywords:** *Adolescent*, *Chen Internet Addiction Scale (CIAS)*, *Internet Addiction*, *Psychological Characteristics*, *Youth Self-Report (YSR)*

## Abstract

**Objective:** This study aimed to measure the prevalence of internet addiction and its impact on the psychological well-being of adolescents in Tehran, considering the sociodemographic characteristics.

**Method** **:** In this cross sectional study, a total of 945 (mean age of 14.85) students (522 boys and 423 girls) were recruited by 2-stage clustering sampling method in 2017. The Chen Internet Addiction Scale (CIAS) and Youth Self-Report (YSR) were used to measure internet addiction and psychological characteristics, respectively. The data were analyzed using multiple-logistic regression analysis adjusted for internet addiction and sociodemographic variables.

**Results: **Overall, 20% of the adolescents were internet addicts. Gender, consanguineous marriage, and father’s education level were significantly associated with internet addiction. Regarding emotional and behavioral characteristics, internet addiction was significantly associated with the scores of internalizing (OR = 5.03; 95% CI: 3.05-8.28) and externalizing problems (OR = 5.84; 95% CI: 3.61-9.43), the total score of empirical scales (OR = 6.51; 95% CI: 3.71-11.6), and all DSM-oriented scales of the YSR (p < 0.001). Except for school performance, other competency scales had no correlations with internet addiction.

**Conclusion: **Regarding the high prevalence of the internet addiction and its correlation with emotional and behavioral characteristics, students and their parents should be advised of the detrimental impacts of internet addiction and try to focus on its constructive application.

Adolescence is a period of development during which teenagers experience major biological, psychological, and social changes. Adolescents are, hence, challenged with managing a variety of existing and emerging health concerns and problems (1). Emotional and behavioral characteristics of adolescence can be evaluated through viewpoints of parents, adolescents themselves, or friends and teachers (2). Based on these viewpoints, several standard subjective methods, in the form of questionnaires, have been developed to measure the behaviors of the adolescents. These assessment tools facilitate screening and comparisons by determining cutoff points for distinguishing healthy and problematic behaviors. The application of these questionnaires also makes it easier to run evaluations on a high number of samples in the society. 

Consequently, various questionnaires, eg, Symptom Checklist-90-Revised (SCL-90-R), General Health Questionnaire (GHQ), Brief Symptoms Inventory (BSI), DePaul's ADHD (attention-deficit/hyperactivity disorder), and Youth Self-Report (YSR), have been used to assess mental illnesses and psychiatric disorders (3-6).

While some common problems related to the adolescence period, including gambling, alcohol abuse, and smoking have been assessed in previous studies (7, 8), a new phenomenon, eg, internet addiction has emerged in recent years. The internet has become one of the most important academic and recreational tools for adolescents and adults. It provides easy and immediate access to information and facilitates communication. However, failure to control internet use may lead to adverse effects on daily life function, family relationships, and emotional stability (9, 10). Various coexisting psychiatric disorders such as major depressive disorder, social anxiety, hostility, aggressive disorder, attention deficit, and ADHD are also attributed to internet addiction (11). Internet addiction has been reported not only in Western but also Eastern societies (12, 13). Although internet addiction has not yet been incorporated into the Diagnostic and Statistical Manual of Mental Disorders (DSM), internet gaming is included as an excessive behavioral problem in the substance-related and addictive disorders section of the DSM-5 (14). Nevertheless, during the recent years, increasing attention has been paid to internet addiction, and standard scales have been proposed for its measurement. Efforts to introduce diagnostic criteria for this condition have resulted in the development of internet addiction scales such as Internet Addiction Test (IAT) and Chen Internet Addiction Scale (CIAS), (15). In recent years, some related instruments were translated and psychometrically evaluated in Iran (16-18). According to a systematic review by Kuss et al, the prevalence of internet addiction ranges from 0.8% in Italy to 26.7% in Hong Kong(15). According to a meta-analysis by Modara et al in 2017, the overall prevalence of internet addiction in Iran was 20% [95% confidence interval (CI): 16%-25%] The meta regression model showed that the trend of internet addiction growth rate in Iran increased from 2006 to 2015 (19). In a nationwide study in Iran, 4342 high school or precollege schools students were recruited from 13/31 provinces of Iran; and 22.2% of the study participants were labeled as having “internet addiction" (20). A previous study in an Iranian society in 2007 showed that about half of the high school students use the internet and about 3.8% are internet addicts. The study also indicated that internet addicted adolescents were lonelier and had lower self-esteem (21).

In addition to internet addiction, some sociodemographic and economic factors may affect psychological well-being of the addicts. Having knowledge about the particular roles and impacts of these underlying factors in the population is essential for obtaining a comprehensive understanding of the main causes of psychological disorders, which in turn facilitates the prevention and treatments. Furthermore, since psychological status can affect some of the mentioned factors, including addictions, the relationship between these factors and psychological conditions should be considered as a mutual pathway rather than a simple cause and effect association (11). Previous studies showed that psychological well-being is inferred differently by children and their parents and teachers. Moreover, it was shown that cultural differences could cause discrepancies between the results (22, 23). Therefore, in this study, firstly, the prevalence of internet addiction in Iranian adolescents was assessed using self-reports of students; then, the relationship between internet addiction and emotional and behavioral problems of adolescents was evaluated considering sociodemographic variables using Youth Self-Report (YSR) Questionnaire to have a comprehensive insight on both empirical and DSM scales. Furthermore, the impact of internet addiction on competency scales was investigated.

## Materials and Methods

***Samples***

This cross sectional study was performed during March-August, 2017. All 22 districts of Tehran (Iran) were categorized as 5 geographical areas of north, south, west, east, and center. The sampling method of the study was 2-stage clustering method. Five girls and five boys’ high schools were randomly selected. Classes of seventh, eighth, ninth, tenth, and eleventh grades (12-18 years old) were randomly selected in each school and data of all students in each class were collected. After being provided with explanations about the study objectives and relevant instructions, the selected students were asked to sign an informed consent form. From the 1204 interviewed students, 1131 had access to the internet (whether at school or home) from whom, 945 students (522 girls and 423 boys), with the mean age of 14.58 years, participated in the study and filled out all the questionnaires.

***Sample Size Calculation***

The sample size was calculated based on multiple-logistic regression model. A small size effect of 0.015, at significance level of α = 0.05, and a statistical power of 1-β = 0.95, was also considered. Based on these inputs, a sample size of 942 subjects was obtained using G*Power 3.0.1 software (24).

***Ethical Approval***

The study protocol was reviewed and approved by the Clinical Research Ethics Board of Tehran University of Medical Sciences, Tehran, Iran.

***Instruments and Questionnaires***

The validated Persian version of the Youth Self-Report (YSR) and Chen Internet Addiction Scale (CIAS) (25, 26) was administrated for evaluating psychological disorders and internet addiction, respectively. A researcher-made questionnaire was also used to collect demographic characteristics. The paper version of the questionnaires was used for all the evaluations.

***Demographic Questionnaire***

A researcher-made demographic questionnaire was used to collect data on gender, age, parents’ age, education levels, jobs, consanguineous marriage, history of receiving counseling services, psychiatric service, birth order, number of siblings, and marital status.

***The Youth Self-Report (YSR)***

The YSR was completed by the adolescents who participated in this study. The YSR is a 112-item scale which scores behavioral problems experienced during the past 6 months on a 3-point Likert scale (0 = not true, 1 = sometimes or somewhat true, and 2 = very true or often true). The scores are used to calculate some summary variables and psychological problem-related scales, including 8 empirically derived syndrome scales. These scales are then categorized into 2 higher order factors (internalizing and externalizing problems). Moreover, there are 6 DSM-oriented scales, eg, depression, anxiety, somatic complains, oppositional defiant disorder (ODD), attention deficit hyperactivity disorder (ADHD), and conduct problems. The YSR also measures 3 competency scales, including activities, school performance, social relations, and their total score (2). The Persian Youth Self-Report scale had satisfied internal consistency, test-retest reliability, and concurrent and construct validity (25).

***The Chen Internet Addiction Questionnaire (CIAS)***

The CIAS is a 26-item questionnaire designed to assess internet addiction (4). The items are scored on a 4-point Likert scale from 1 (strongly disagree) to 4 (strongly agree). The scores range between 26 and 104, with higher scores indicating the higher severity of internet addiction. The scores between 26-63, 64-67, and 68-104 suggest normal, being at-risk, and internet addicted individuals, respectively (27). In this study, due to the small number of individuals in the risk group, it was merged with the normal group, and the cutoff point was set at 68. Moreover, the CIAS can evaluate 5 domains, including compulsive use, withdrawal, tolerance, problems in interpersonal relations, and time management problems (28). The Chen Internet Addiction Scale had intense convergent validity (r = 0.85) and high internal consistency (α = 0.93) (26).

***Statistical Analyses***

Data were presented as mean ± standard deviation (SD) for quantitative variables and as frequency and percentage for qualitative variables. The mean, SD, and range of scores were computed for the CIAS. Chi-square test was used to evaluate the relationships between internet addiction and some demographic variables. However, as these variables had p values less than 0.20, a multiple-logistic regression analysis was performed, and 4 sets of models were constructed. Akaike Information Criterion (AIC) was applied to compare the models to identify the most important demographic predictors of internet addiction. In the first model, age and gender were included as predicting variables. Parents’ levels of education were then added to the second model. The third model also included counseling services. Finally, consanguineous marriage was incorporated into the fourth model. Internet addiction was the dependent variable in all models. Eventually, based on the least AIC, the best model (Model 4) was selected for evaluation. Moreover, for all variables in each model, odds ratios (ORs), 95% confidence intervals (CIs), and p values were calculated.

Associations between each subgroup of the YSR scales and internet addiction were estimated based on chi-square tests. Spearman’s correlations between the subgroups of the YSR and internet addition were calculated for each gender. Also, to identify the relationships between the YSR and internet addition, 3 multiple-logistic regression models were conducted for each subgroup of the YSR (empirical scales, DSM, competency). Age, gender, and internet addiction were inserted in all models as the predicting variables. For other variables, p values were calculated using chi-square, and variables with p values less than 0.2 were entered into the model (29, 30). For all variables in each model, ORs and 95% confidence interval were calculated.

## Results

Based on the descriptive analysis, 20% (189 out of 945) of the participants had symptoms of internet addiction (based on cutoff point of 68 set for this study) in the past 3 months. According to the results of thechi-square test, except for consanguineous marriage (p = 0.04), other sociodemographic variables (gender, age, mother’s education, father’s education, and history of psychological counseling) were not significantly different between nonproblematic and internet-addicted groups (Table 1). However, as these nonsignificant variables had p values less than 0.20, a multiple-logistic regression analysis was performed, and the Akaike Information Criterion (AIC) was applied for all models. The results were obtained from the best model (Model 4), which showed that after separating the role of each variable, gender (odds ratio (OR) = 1.43; P = 0.047), consanguineous marriage (OR = 1.56; P = 0.035), and father’s education (not holding high school diploma, OR = 1.78; P = 0.044) were associated with internet addiction (Table 2).

Nonproblematic and internet-addicted students were significantly different regarding all problem-related (DSM-oriented and empirical) scales of the YSR (P < 0.001). Internet addiction was also significantly related with total competency (p = 0.038) and school performance (p < 0.001; Table 3). According to the results of multiple-logistic regression analysis for predicting scores obtained from the YSR scales, after adjustment for sociodemographic characteristics (gender, age, father’s education, mother’s education, consanguineous marriage, history of psychological counselling, birth order, number of siblings, father’s job, mother’s job, marital status, father’s age, and mother’s age), internet addiction had significant effects on all problems related to subscales of the YSR (empirical and DSM-oriented). Internet-addicted students scored significantly higher in all DSM-oriented and empirical scales (p < 0.001). The highest effect belonged to total scores of empirical scales (OR = 6.51), followed by conduct problems (OR = 6.10), (Tables 4, 5). On the other hand, while internet addiction lost its impact on total competency scale after adjustment for sociodemographic characteristics (P = 0.18), it was still associated with school performance (OR = 2.09) (Table 6).Age had small effects on problem-related scales of the YSR, eg, it had significant effects on only 2 subscales (externalization problems and ODD), (Table 4, 5). However, its effect on the total competency scale was substantial, as it impacted all its 3 subscales (p < 0.05, Table 6). Moreover, despite its greater effects (compared to age), gender had no unanimous effects on problem-related YSR scales as well (Tables 4, 5). However, similar to age, the effect of gender was relatively higher on the competency scales, including school performance (OR = 11.15), followed by total competency score (OR = 5.34), which were significantly related to gender (Table 6).

**Table 1:** Relationship between Internet Addiction and Socio-Demographic Variables

**parameters**	**Sample**	**Internet addiction ** **(CIAS)**		**P value**
		**Clinical**	**Normal**	
	**%(n)**	**%(n)**	**%(n)**	
**Total**	100(945)	20(189)	80(756)	
**Sex**				0.113
Girl	55.27(522)	21.7(113)	78.3(409)	
Boy	44.73(423)	17.6(74)	82.4(349)	
**Age (years)**				0.205
12-14	42.6(403)	18.1(73)	81.9(330)	
15-18	57.4(542)	21.5(117)	78.5(725)	
**Father Education**				0.05
Under Diploma	25.5(241)	25.8(62)	74.2(179)	
Diploma College Education	34.8(329) 39.7(375)	20.3(67) 17.4(65)	79.7(262) 82.6(310)	
**Mother Education** Under Diploma Diploma College Education **Consanguineous ** **marriage**	22.9(217) 43.2(408) 33.9(320)	24.7(54) 19.3(79) 18.4(59)	75.3(163) 80.7(329) 81.6(261)	0.186 0.04
No family relationship Family relationship	74.1(700) 25.9(245)	21.7(152) 15.5(38)	78.3(548) 84.5(207)	
**consulting service** Yes No	24.7(233) 75.3(712)	22.9(53) 19(135)	77.1(180) 81(577)	0.191

CIAS: Chen Internet Addiction Scale

**Table 2:** Association between Internet Addiction (CIAS) and Sociodemographic Variables Based on Multiple Logistic Regression Model

**Parameters**	**Model 1**		**Model 2**		**Model 3**		**Model 4**	
	**OR (95% CI)**	**P value**	**OR (95% CI)**	**P value**	**OR (95% CI)**	**P value**	**OR (95% CI)**	**P value**
**Sex**								
Girl	1.30 (0.93,1.80)	0.113	1.44 ( 1.02,2.04)	0.038	1.45 (1.02,2.05)	0.037	1.43 (1.004,2.04)	0.047
Boy(ref.)	1.00		1.00		1.00		1.00	
**Age(years)**								
12-14	0.80 (0.58,1.12)	0.201	0.83 (0.58,1.18)	0.304	0.83 (0.58,1.18)	0.300	0.82 (0.57,1.17)	0.276
15-18(ref.)	1.00		1.00		1.00		1.00	
**Mother ** **Education**								
Under Diploma			1.04 (0.57,1.86)	0.902	1.04 (0.58,1.87)	0.888	1.10 (0.60,1.99)	0.754
Diploma			0.86 (0.55,1.36)	0.532	0.86 (0.55,1.36)	0.545	0.85 (0.54,1.36)	0.514
College Education (ref.)			1.00		1.00		1.00	
**Father ** **Education**								
Under high school diploma			1.73 (0.99,3.01)	0.052	1.79 (1.02,3.12)	0.040	1.78 (1.01,3.13)	0.044
Diploma			1.29 (0.82,2.04)	0.264	1.32 (0.84,2.10)	0.223	1.30 (0.819,2.07)	0.263
College Education (ref.)			1.00		1.00		1.00	
**Consulting ** **service**								
Yes					1.26 (0.86,1.86)	0.228	1.30 (0.88,1.93)	0.179
No(ref.)					1.00		1.00	
**Consanguin** **eous ** **marriage**								
No family relationship							1.56 (1.03,2.37)	0.035
family relationship(r ef.)							1.00	
**AIC**	930.17		844.60		838.91		817.93	

CIAS: Chen Internet Addiction Scale; AIC: Akaike's Information Criterion

**Table 3:** Association between Internet Addiction (CIAS) and Youth Self-Report (YSR)

**YSR**		**Internet addiction (CIAS)**		**P value**
		**Clinical** **n=189** **(mean±SD)**	**Normal** **n=756** **(mean±SD)**	
Empirically scales	Externalizing problems	10.91±6.69	18.87±8.55	<0.001
	Internalizing problems	13.08±8.11	20.41±9.40	<0.001
	Total problems	38.08±19.68	62.59±23.57	<0.001
DSM based scales	Affective problems	5.75±3.91	9.03±4.44	<0.001
	Anxiety problems	2.49±1.79	3.40±2.07	<0.001
	Somatic problems	1.69±1.97	2.68±2.51	<0.001
	ODD	2.81±2.13	4.71±2.27	<0.001
	ADHD	4.05±2.59	5.94±2.82	<0.001
	Conduct problems	3.66±3.24	7.47±4.52	<0.001
Competence scales	Activity	8.61±3.43	8.37±3.35	0.848
	Social relations	10.43±2.95	10.18±2.73	0.455
	School performance	2.24±0.54	2.04±0.58	<0.001
	Total score	20.16±5.23	19.39±4.97	0.038

YSR: Youth Self-Report; CIAS: Chen Internet Addiction Scale; DSM: Diagnostic and Statistical Manual of Mental Disorders; ODD: Oppositional Defiant Disorder; ADHD: Attention Deficit Hyperactivity Disorder

**Table 4:** Relationship between YSR (Empirically Scales) and Internet Addiction (CIAS) Adjusted on Sociodemographic Variables

	**Externalizing problems**	**Internalizing problems**	**Problem behavior total**
	**Odds Ratio** **(95% CI)**	**Odds Ratio** **(95% CI)**	**Odds Ratio** **(95% CI)**
**Gender** Girls Boys(ref.)	2.03 (1.23-3.34) 1.00	2.00 (1.21-3.31) 1.00	1.17 (0.69-1.98) 1.00
**Age(years)** 15-18 12-14(ref.)	1.70 (1.01-2.87) 1.00	1.15 (0.70-1.88) 1.00	1.56 (0.89-2.72) 1.00
**Father Education** Under Diploma Diploma College Education(ref.) **Birth** **order** Third child and after Second child First child(ref.) **consulting service** Yes No(ref.) **Psychiatry service** Yes No(ref.) **Internet addiction** Clinical Normal(ref.)	2.32 (1.21-4.11) 1.80 (1.01-3.22) 1.00 2.00 (1.21-4.11) 1.00 5.84 (3.61-9.43) 1.00	0.48(0.25-0.91) 0.55 (0.31-0.96) 1.00 2.14 (0.93-4.91) 1.00 5.03 (3.05-8.28) 1.00	2.32 (1.119-4.53) 0.80 (0.43-1.48) 1.00 3.58 (1.40-9.17) 1.00 6.51(3.71-11.60) 1.00

YSR: Youth Self-Report; CIAS: Chen Internet Addiction Scale

**Table 5:** Relationship between YSR (DSM Based Scales) and Internet Addiction (CIAS) Adjusted on Sociodemographic Variables

	**Affective ** **problems**	**Anxiety ** **problems**	**Somatic ** **problems**	**ODD ** **Problems**	**ADHD ** **problems**	**Conduct ** **problems**
	**Odds Ratio** **(95% CI)**	**Odds Ratio** **(95% CI)**	**Odds Ratio** **(95% CI)**	**Odds Ratio** **(95% CI)**	**Odds Ratio** **(95% CI)**	**Odds Ratio** **(95% CI)**
**Gender**						
Girls	1.86 (1.22-1.28)	1.80 (1.06-3.04)	0.92 (0.61-1.40)	1.79 (1.21-2.63)	1.43 (0.88-2.31)	2.12 (1.38-3.26)
Boys(ref.)	1.00	1.00	1.00	1.00	1.00	1.00
**Age(years)**						
15-18	1.19 (0.79-1.80)	0.78 (0.48-1.27)	0.85 (0.56-1.30)	1.74 (1.17-2.59)	1.31 (0.81-2.12)	1.31 (0.86-1.99)
12-14(ref.)	1.00	1.00	1.00	1.00	1.00	1.00
**Mother Education**						
Under Diploma	1.12 (0.70-1.79)					1.80 (1.08-2.99)
Diploma	1.68 (1.00-2.85)					2.37 (1.32-4.23)
College Education(ref.)	1.00					1.00
**Family status**						
Divorced	2.17 (1.07- 4.40)					
Together(ref.)	1.00					
**Father job**						
Without Job	1.70 (0.81-3.53)	2.34 (1.02-5.39)				
Working(ref.)	1.00	1.00				
**Mother job**						
Without Job						0.47 (0.28-0.79)
Working(ref.)						1.00
**consulting service**						
Yes		1.71 (1.02-2.81)	2.63 (1.71-4.07)			
No(ref.)		1.00	1.00			
**Psychiatry service**						
Yes	3.16 (1.51-6.60)			2.64 (1.27-5.48)	3.34 (1.57-7.04)	2.66 (1.25-5.64)
No(ref.)	1.00			1.00	1.00	1.00
**Consanguineous ** **marriage**						
No family relationship		1.18 (0.66-2.11)		1.44 (0.90-2.29)	1.92 (1.04-3.56)	
Family relationship(ref.)		1.00		1.00	1.00	
**Internet addiction**						
Clinical	3.89 (2.56-5.93)	2.43 (1.45-4.06)	2.34 (1.48-3.71)	4.79 (3.18-7.22)	3.72 (2.33-5.95)	6.10 (4.03-9.23)
Normal(ref.)	1.00	1.00	1.00	1.00	1.00	1.00

YSR: Youth self-report; DSM: Diagnostic and Statistical Manual of Mental Disorders; CIAS: Chen Internet Addiction Scale; ODD: Oppositional Defiant Disorder ;ADHD: Attention Deficit Hyperactivity Disorder

**Table 6:** Relationship between YSR Competence Scales and Internet Addiction (CIAS) Adjusted on Sociodemographic Variables

	**Activity**	**Social relations**	**School performance**	**Competence scales**
	**Odds Ratio** **(95% CI)**	**Odds Ratio** **(95% CI)**	**Odds Ratio** **(95% CI)**	**Odds Ratio** **(95% CI)**
**Gender**				
Girls	1.04 (0.66-1.65)	0.51 (0.34-0.76)	11.15 (3.93-31.61)	5.34 (3.84-7.41)
Boys(ref.)	1.00	1.00	1.00	1.00
**Age(years)**				
15-18	2.44 (1.46-4.09)	1.56 (1.03-2.36)	3.61 (1.69-7.69)	2.19 (1.58-3.04)
12-14(ref.)	1.00	1.00	1.00	1.00
**Father Education**				
Under Diploma			1.67 (0.80-3.47)	1.64 (1.13-2.38)
Diploma			1.92 (0.87-4.24)	1.62 (1.07-2.47)
College Education(ref.)			1.00	1.00
**Mother ** **age(years)**				
More than 50		0.77 (0.28-2.13)		
Between 40 and 50		0.48 (0.56-1.31)		
Less than 40(ref.)		1.00		
**Mother job**				
Without Job				1.60 (1.06-2.41)
Working(ref.)				1.00
**Birth** **order**				
Third child and after	1.80 (0.98-3.28)			
Second child	1.23(0.73-2.06)			
First child(ref.)	1.00			
**Number of ** **children**				
4 and more		0.29 (0.14-0.62)		0.28 (0.14-0.54)
3		0.32 (0.17-0.58)		0.43 (0.24-0.74)
2		0.25 (0.15-0.42)		0.42 (0.26-0.69)
1(ref.)		1.00	1.00	1.00
**consulting ** **service**				
Yes			2.74 (1.49-5.04)	0.62 (0.432-0.90)
No(ref.)			1.00	1.00
**Internet addiction**				
Clinical	0.97(0.55-1.72)	1.09 (0.67-1.77)	2.09 (1.11-3.96)	1.31 (0.88,1.95)
Normal(ref.)	1.00	1.00	1.00	1.00

YSR: youth Self-Report; CIAS: Chen Internet Addiction Scale

## Discussion

***Prevalence of Internet Addiction***

The amount of time spent online on the internet has gradually increased in recent years. Despite its several benefits, internet use can become detrimental if it is not adequately controlled (31). This study evaluated the prevalence of internet addiction and its probable relationships with psychological disorders and sociodemographic variables among Iranian adolescents. This 20% prevalence of internet addiction in the Iranian society is more than that in most European and Asian 

countries. Different studies have administered a variety of instruments with different criteria and cutoff points for measuring the prevalence of internet addiction. Moreover, studies have been conducted in different times and internet addiction has an increasing trend with time. Therefore, a precise comparison between their results may not be plausible. Nevertheless, the reported prevalence rates ranged between 0.8% and 26.7% (15); and the obtained prevalence in this study stood high in this range. In fact, the prevalence of internet addiction in the present study was a little higher than other studies that also administered the CIAS for the assessment of internet addiction (20.0% vs. 10.8%, 18.8%, 12.3%, and 17.1%), which were performed in Taiwan (32-35). The obtained rate was also higher than most European countries (13.9%), China (15.2%), and Japan (2% addicted, 21.7% possibly addicted), where the IAT seems to be the most popular instrument for internet addiction measurement (6, 36, 37).

A previous study which took place in Iran (21) reported the prevalence of 3.8% for internet addiction among Iranian high school students. However, in that study, the administrated instrument (Internet Addiction Test (IAT)) to determine internet addiction was different from this study (Chen Internet Addiction Scale (CIAS)). However, compared to that study, the rate of 20% for internet addicts among Iranian adolescents in 2017 is significant. Other studies in the Iranian society mostly focused on specific groups of medical students. Therefore, they cannot be representative of the general population (27, 28). The high and increased prevalence of internet addiction in this study may be attributed to the fact that Iran is a developing country, where now compared with a decade ago, many young individuals have access to smartphones and the internet. In addition, cultural differences may play an important role in this regard. This study was conducted in the capital of Iran, and the availability of the required resources may have caused such a high prevalence. Moreover, Internet-based social networks have emerged over the past decade which have attracted the attention of many adolescents. This significant increase in internet use and addiction may also imply a cultural change and the move from individualistic to a collective society, where socializing is important (21). 

In contrast to the results of most European and Asian studies (6, 15, 32, 38)(15, 28, 34, 35), female participants in this study were more addicted to the internet. The results of this study are also in contrast with the results of a study on medical students in Mashhad, Iran (27). The higher amount of internet use among girls in this study can be justified by the fact that Iranian teenage girls are less involved in outdoor activities than their male counterparts; therefore, they have a higher tendency for exploring the internet in their free time. There were no differences between younger and older adolescents regarding internet addiction in this study, which is in line with a previous report (15). As mentioned in previous research, the effect of the first encounter with the internet is important in the level of internet addiction and related side-effects (39). To the best of the authors’ knowledge, the first encounter of the students in this study had already occurred since the internet is widely accessible at least in schools. In this study, the adolescents whose fathers were less educated and their parents had consanguineous marriage were more addicted to the internet, which may be due to the less motivations by these parents to be involved in children's education (40). Two other studies also reported the higher frequency of internet addiction among adolescent with a lower educational level of parents (6, 41). 

***Emotional and Behavior Characteristics of Internet Addict and Nonaddict Adolescents***

Evaluating the psychosocial characteristics of the students revealed the coexistence of psychological problems and internet addiction. All the YSR scales measured in this study (eg, externalizing problems, internalizing problems, total problems of empirical scales, and DSM-oriented scales, including depression, anxiety, somatic complains, oppositional defiant disorder (ODD), attention deficit hyperactivity disorder (ADHD), and conduct problems) had higher scores in internet addicts. Using different scales and instruments with different criteria and cutoff points, previous studies also showed the association between psychological disorders and internet addiction (6, 11, 31, 35, 39)). In this study, the association between internet addiction and problem-related YSR scales was so strong that the comorbidity was still present even after adjustment for sociodemographic variables. Studies that assessed psychological disorders with instruments other than the YSR have also indicated strong associations between internet addiction and psychological disorders. Kawabe et al, for instance, showed that the total score of the General Health Questionnaire and the scores of its subscales were strongly associated with problematic internet use in junior high school students, especially those who used smartphones more frequently (37).

Among competency scales, total competency score and school performance were associated with internet addiction. However, total competency score lost its significance after adjustment for sociodemographic variables. The results of this study differed from Artemis' studies in some competency scales but agreed in others. Artemis showed internet-addicted adolescents had lower scores in competency scales, except for social competency (6). In another study, Artemis showed a higher social competency in older adolescents who use social networking sites heavily (39). The negative effects of internet addiction on school performance in this study can be explained by the fact that spending more time online decreases adolescents’ tendency for offline activities that are the sources of rewards (42). Also, more time on the internet means less time for doing homework and learning.

After internet addiction, gender showed the strongest association with psychological disorders and affected all problem-related YSR scales except somatic complains, ADHD, and total behavioral problems. However, age was only associated with 2 problem-related scales, eg, externalizing problems and ODD. Unlike problem-related scales, all competency scales were properly predicted by age. Gender could also strongly predict competency scales, except for activity and social relations. Overall, female gender, older age, and internet addiction worsened school performance, ODD, and externalizing problems. 

To the best of the authors’ knowledge, this was the first study to investigate the relationship between internet addiction and emotional and behavioral characteristics of adolescents using YSR scales.

## Limitation

The findings of this study were obtained within the limitations of its constructed model. To draw a comprehensive conclusion regarding the association between psychological disorders and internet addiction, future studies are recommended to investigate factors such as tobacco abuse and gambling problems in addition to sociodemographic variables. Furthermore, temporal relations between internet addiction and psychological disorders as well as the potential role of internet addiction in the incidence of psychological disorders have not yet been determined. It should also be clarified whether treating psychological disorders can reduce internet addiction and vice versa. Longitudinal and cohort designs are suggested for future studies in this field.

## Conclusion

This study represented the high prevalence of 20% internet addicts among Iranian adolescents, which is more than most European and Asian countries. Moreover, internet addiction was associated with all the problem-related scales of YSR. Competency scales, except for school performance, were not associated with internet addiction. Female gender, older age, and higher internet addiction worsened school performance, ODD, and externalizing problems. Therefore, the simultaneous treatment of both internet addiction and psychological disorders is highly recommended.
